# *Schistosoma haematobium* infection and environmental factors in Southwestern Tanzania: A cross-sectional, population-based study

**DOI:** 10.1371/journal.pntd.0008508

**Published:** 2020-08-24

**Authors:** Kirsi M. Manz, Inge Kroidl, Petra Clowes, Martina Gerhardt, Wilbrod Nyembe, Lucas Maganga, Weston Assisya, Nyanda E. Ntinginya, Ursula Berger, Michael Hoelscher, Elmar Saathoff

**Affiliations:** 1 Institute for Medical Information Processing, Biometry, and Epidemiology (IBE), Ludwig-Maximilians University, Munich, Germany; 2 Division of Infectious Diseases and Tropical Medicine, University Hospital, LMU Munich, Germany; 3 NIMR-Mbeya Medical Research Center (MMRC), Mbeya, Tanzania; 4 German Center for Infection Research (DZIF), partner site Munich, Munich, Germany; Swiss Tropical and Public Health Institute, SWITZERLAND

## Abstract

Schistosomiasis is a leading cause of morbidity in Africa. Understanding the disease ecology and environmental factors that influence its distribution is important to guide control efforts. Geographic information systems have increasingly been used in the field of schistosomiasis environmental epidemiology. This study reports prevalences of *Schistosoma haematobium* infection and uses remotely sensed and questionnaire data from over 17000 participants to identify environmental and socio-demographic factors that are associated with this parasitic infection. Data regarding socio-demographic status and *S*. *haematobium* infection were obtained between May 2006 and May 2007 from 17280 participants (53% females, median age = 17 years) in the Mbeya Region, Tanzania. Combined with remotely sensed environmental data (vegetation cover, altitude, rainfall etc.) this data was analyzed to identify environmental and socio-demographic factors associated with *S*. *haematobium* infection, using mixed effects logistic regression and geostatistical modelling. The overall prevalence of *S*. *haematobium* infection was 5.3% (95% confidence interval (CI): 5.0–5.6%). Multivariable analysis revealed increased odds of infection for school-aged children (5–15 years, odds ratio (OR) = 7.8, CI: 5.9–10.4) and the age groups 15–25 and 25–35 years (15–25 years: OR = 5.8, CI: 4.3–8.0, 25–35 years: OR = 1.6, CI: 1.1–2.4) compared to persons above 35 years of age, for increasing distance to water courses (OR = 1.4, CI: 1.2–1.6 per km) and for proximity to Lake Nyasa (<1 km, OR = 4.5, CI: 1.8–11.4; 1–2 km, OR = 3.5, CI: 1.7–7.5; 2–4 km; OR = 3.3, CI: 1.7–6.6), when compared to distances >4 km. Odds of infection decreased with higher altitude (OR = 0.7, CI: 0.6–0.8 per 100 m increase) and with increasing enhanced vegetation index EVI (OR = 0.2, CI: 0.1–0.4 per 0.1 units). When additionally adjusting for spatial correlation population density became a significant predictor of schistosomiasis infection (OR = 1.3, CI: 1.1–1.5 per 1000 persons/km^2^) and altitude turned non-significant. We found highly focal geographical patterns of *S*. *haematobium* infection in Mbeya Region in Southwestern Tanzania. Despite low overall prevalence our spatially heterogeneous results show that some of the study sites suffer from a considerable burden of *S*. *haematobium* infection, which is related to various socio-demographic and environmental factors. Our results could help to design more effective control strategies in the future, especially targeting school-aged children living in low altitude sites and/or crowded areas as the persons at highest need for preventive chemotherapy.

## Author summary

Urinary schistosomiasis caused by the parasitic blood fluke *Schistosoma haematobium* is an important public health concern in the tropics and subtropics. Freshwater snails of the genus *Bulinus* are the intermediate hosts and infection is acquired by contact with fresh water inhabited by infected snails. This means that transmission of the parasite strongly depends on environmental factors, which we wanted to explore in this study. Individually assessed questionnaire and laboratory data from a study in Mbeya Region in Southwestern Tanzania were combined with remotely sensed and other environmental data that were publicly available. We found that the odds of urinary schistosomiasis were higher in school-aged children and young adults than in older age groups and in children below 5 years. Odds of infection were also higher at lower altitudes, in areas with less vegetation and in the vicinity of Lake Nyasa. These results add to the general knowledge regarding *S*. *haematobium* infection and could help to design more effective control strategies in the future, both locally and on larger scales.

## Introduction

Trematodes of the genus *Schistosoma* are among the most common infectious agents of humans. They cause schistosomiasis which occurs in 78 tropical and sub-tropical countries. The highest prevalences are encountered in Sub-Saharan-Africa (SSA), where according to recent estimates there are 112 million cases of *Schistosoma haematobium* infection and 54 million cases of *Schistosoma mansoni* infection [1]. The current main strategy to combat schistosomiasis in SSA and other developing parts of the world is mass administration of praziquantel to school age children, with the aim to reduce the disease burden. According to WHO, at least 221 million people world-wide and 200 million in Africa required preventive mass-treatment for schistosomiasis in 2017, out of which 102 million world-wide and 91 million people in Africa were reported to have been treated [1].

Schistosomiasis infection occurs through contact with fresh water contaminated with the free-swimming larval forms (cercariae) of the parasite. The cercariae penetrate the skin and mature to adulthood inside the human body. Adult worms live in the veins draining the urinary tract (*S*. *haematobium*) or the intestine (other species). The eggs, which are passed into the environment with the feces or urine, hatch in fresh water and release so-called miracidia, which infect suitable host snails. The snail in turn releases cercariae which infect humans during contact with fresh water. However, many of the eggs are trapped in the human host’s tissues and the body’s reaction to them can cause massive tissue damage. Thus trapped eggs are the main cause of morbidity in schistosome infection [2].

*S*. *haematobium*, the agent causing urinary schistosomiasis, only occurs in Africa and the Middle East, where it is the most common schistosome species [3,4]. Consequences of urinary schistosomiasis include hematuria, dysuria, bladder scarring, chronic urinary tract infection and possibly bladder cancer [5,6]. Up to three-quarters of women with *S*. *haematobium* infection are estimated to suffer from female genital schistosomiasis when eggs accumulate in the vagina, cervix, uterus or fallopian tubes [7–9]. Environmental conditions that are permissive to the development of intermediate host snails are important factors for schistosomiasis transmission in endemic regions. Advances in remote sensing (RS) and geographic information systems (GIS) have enabled researchers to explore these environmental and climatic factors in greater depth. Risk mapping, with the aid of RS and GIS applications, is suited to the study of schistosomiasis as the infectious agents and their snail hosts are sensitive to environmental conditions [10, 11]. When exploring the associations between environmental factors and infection with schistosomiasis the spatial correlation should be taken into account by employing geostatistical models [12–20].

To capture the effects of individual factors and especially the effects of the surrounding environment on the risk of schistosomiasis infection, the situation before any large-scale preventive chemotherapy should be considered. The aim of this study was to report pre-treatment prevalences of *S*. *haematobium* infection and to identify individual factors and local environmental conditions that might influence *S*. *haematobium* transmission in Mbeya Region in Southwestern Tanzania.

## Methods

### Ethics

The EMINI (Evaluating and Monitoring the Impact of New Interventions) cohort study was approved by the ethics committee of the Tanzanian National Institute for Medical Research and conducted according to the Declaration of Helsinki. All participants signed/thumb printed a written informed consent before enrollment into the study, with parents consenting for their minor children below 18 years of age. Children between 12 and 18 years additionally signed/thumb printed the consent document and younger children who were old enough to understand the process were asked to participate in the consenting procedure as well.

### Study area and epidemiological data collection

**Fig 1:**
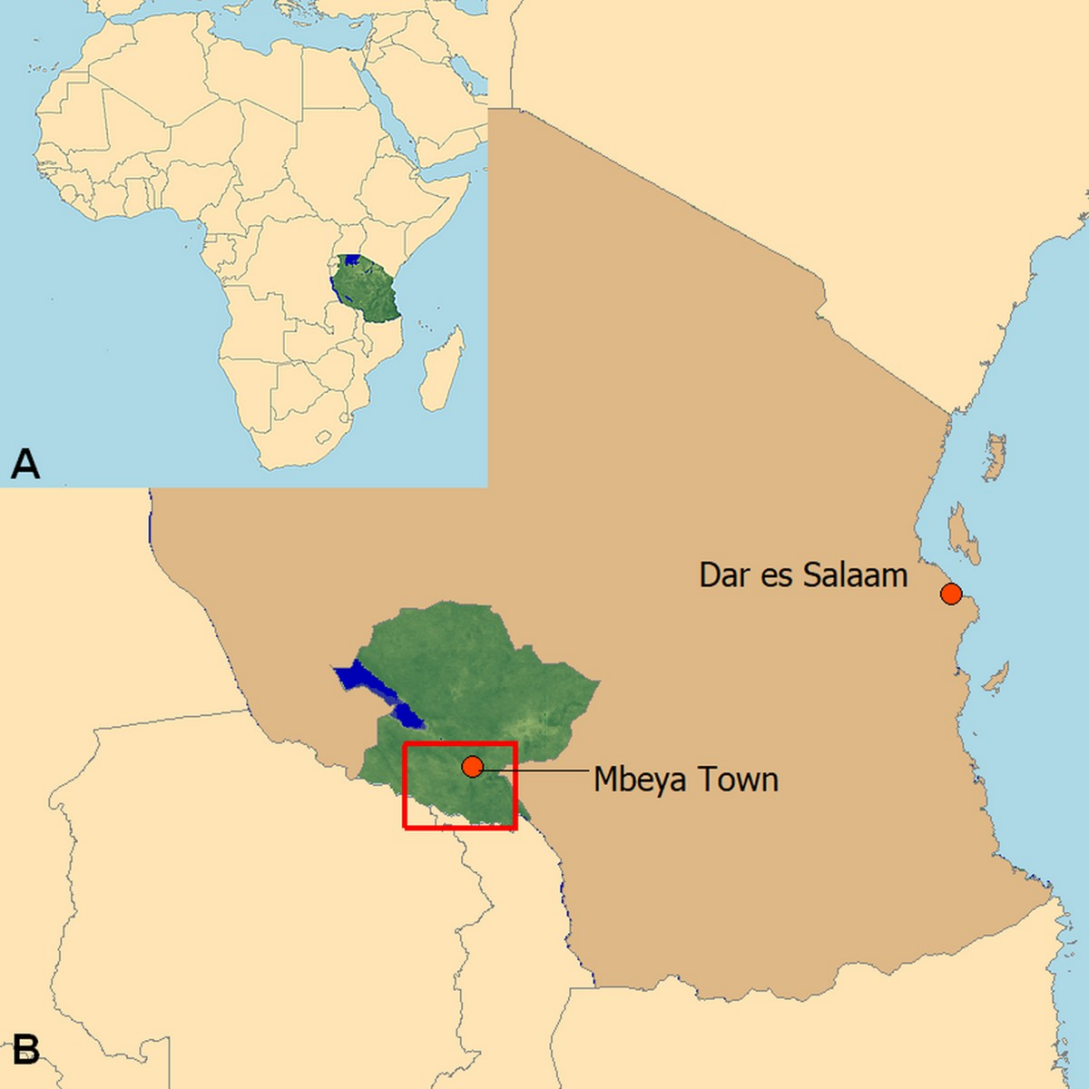
**Map of Africa showing the location of Tanzania (A) and map of Tanzania indicating where Mbeya Region (green area) and the study area (red square) are located (B).** (Original work of the authors).

**Fig 2:**
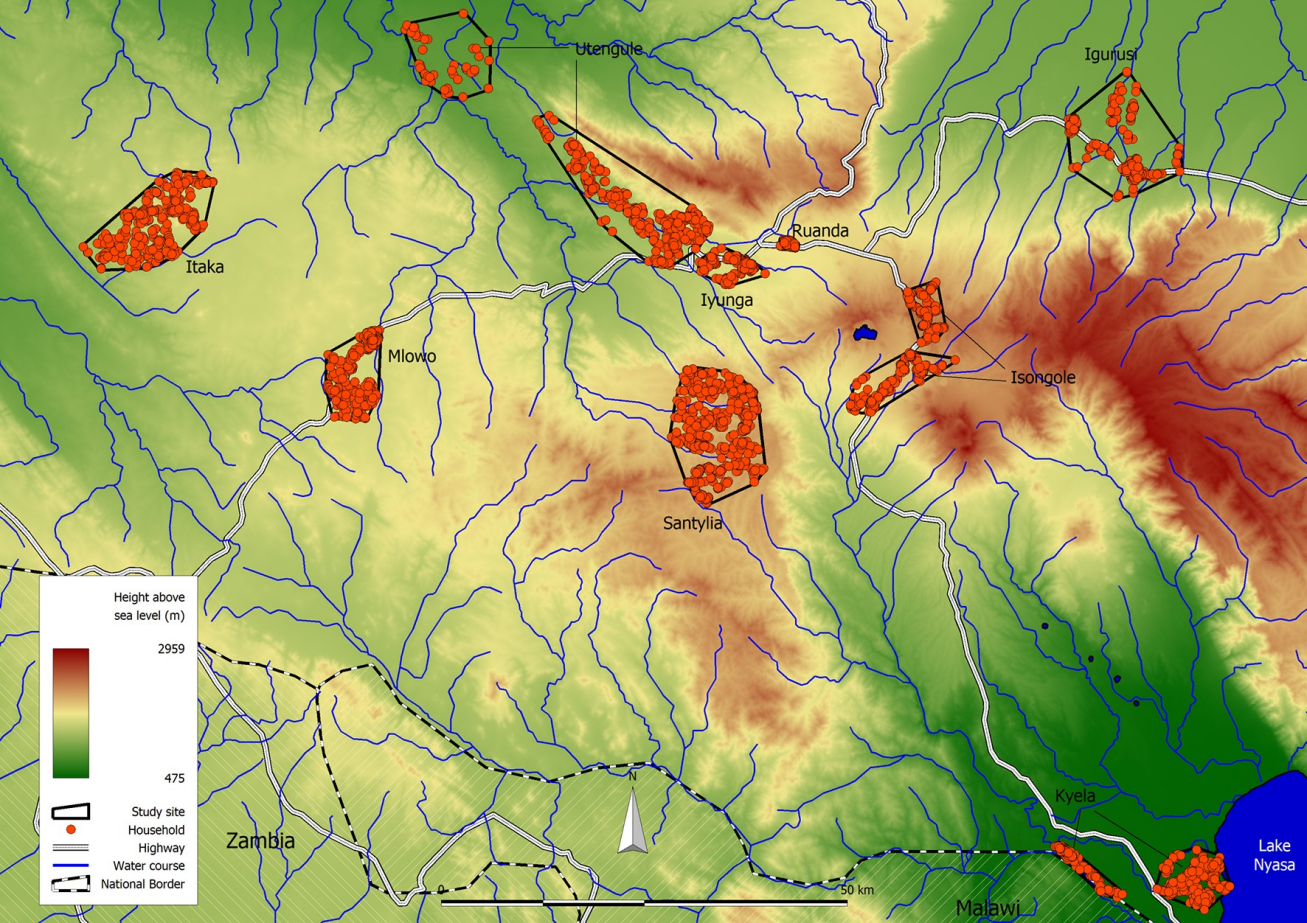
Map of the study area showing the nine distinct study sites. (Original work of the authors).

The study area is located in the Mbeya Region in Southwestern Tanzania and extends from 32.678˚ to 33.963˚ East and from 8.652˚ to 9.649˚ South as shown in Fig 1. Fig 2 provides an overview of the study area, the study sites and the participating households. Below we briefly describe the study area and data collection methods, more detailed accounts and results for other helminth infections in this population are provided elsewhere [21–23].

Prior the start of the EMINI study the study area and the nine study sites were preselected to cover a wide variety of economic and environmental conditions. This was followed by a census of the nine selected sites, covering more than 42,000 households. Household positions were determined using handheld GPS devices. After the census of all households in each site was completed, 10% of the households within these sites were chosen as a geographically stratified random sample to participate in the study. Using the geographical position, each household was visited annually starting in 2006, and all household members who were present during each visit were asked to participate in the study. Within the same annual survey, households were only revisited if the initial visit was completely unsuccessful, i.e. if no household member had been present.

During the survey visits we collected blood and urine samples to test for HIV and schistosomiasis, and performed structured interviews in the local language (Kiswahili). Additional collection of stool samples to study soil-transmitted helminth infections started at the third annual visit from 2008 onwards in 50% of the households. For the analysis of *S*. *haematobium* infection shown here, we used data from the first annual survey which was conducted between May 2006 and May 2007. To our knowledge no larger schistosomiasis control efforts had been implemented in the region prior to this study.

Urine samples were processed as follows: Morning and spot urine samples were collected from each participant and 10 ml of urine from each of the two samples were filtered through polyamide mesh with 20 μm pore size. The filters were stained with Logol’s iodine and microscopically examined for *S*. *haematobium* eggs. All participants with urine samples with at least one *S*. *haematobium* egg were regarded as infected. *S*. *haematobium* infection intensity was recorded in eggs per centiliter (EPC) and classified into no (0 EPC), light intensity (<50 EPC) and heavy intensity infections (≥50 EPC) according to World Health Organization guidelines [24]. Infected participants were offered standard treatment with praziquantel. HIV testing was done using the Determine HIV 1/2 rapid test (Abbott Laboratories, Abbott Park, IL) followed by confirmatory ELISA testing (Enzygnost Anti HIV 1/2 Plus, DADE-Behring, Marburg, Germany) if the RDT was positive. Western blot testing (MPD HIV Blot 2.2, MP Biomedicals, and Geneva, Switzerland) was performed to resolve conflicting results [25].

The financial situation of a household does not necessarily reflect the actual socio-economic status (SES) in low resource settings, particularly because it is difficult to get reliable information on household income and expenditure. To get a more reliable estimate of the SES, we applied a modified method initially proposed by Filmer and Pritchett [26, 27] that uses polychoric principal component analysis to combine different proxies for household wealth into an SES score. The following items were used to construct the score: household belongings (clock or watch, radio, television, mobile telephone, refrigerator, hand cart, bicycle, motor cycle, car, savings account), materials used to build the house, sources of energy and drinking water, number of persons per room. The necessary data were obtained during the household interviews.

### Ecological data

Data regarding the larger lakes in the area and elevation data, which were also used to calculate slope of the terrain, were retrieved from NASA´s Shuttle Radar Topography Mission (SRTM) global digital elevation model, version 2.1 with a nominal resolution of 90 m [28]. Information on water courses was obtained from Vector Map Level 0 (VMAP0) data by the US National Imagery and Mapping Agency. Water courses were defined as elongated flowing water bodies such as streams and rivers, in contrast to ponds or lakes.

Data regarding land surface temperature during the day (LST day) and night (LST night), and green vegetation cover (EVI = enhanced vegetation index) had been collected by NASA’s Moderate-Resolution Imaging Spectroradiometer (MODIS) Terra satellite [29]. LST data (version MOD11A2) have 8 days temporal and about 1 km spatial resolution. Vegetation data (version MOD13Q1) have 16 days temporal and 250 m spatial resolution. Data for the years 2003 through 2008 were retrieved from the online data pool, courtesy of the NASA EOSDIS Land Processes Distributed Active Archive Center (LP DAAC), USGS/Earth Resources Observation and Science (EROS) Center, Sioux Falls, South Dakota (https://lpdaac.usgs.gov/). Both, LST and vegetation data were processed in the following way to produce long-term averages: data surfaces for every 8-day period (LST) and every 16-day period (EVI) for the years 2003 to 2008 were imported into Idrisi GIS software v.32 (Clark Labs, Worcester, MA, United States of America). In Idrisi, annual averages, minima and maxima of day- and night-LST and EVI for each of these six years were calculated for every pixel and in turn used to calculate long term averages for each pixel for the whole period 2003 to 2008. The calculated averages, minima and maxima are thus an average of the six annual minima and maxima for each pixel. For this, we utilized only those pixels that were ‘‘good data quality” according to the quality assessment layers that are distributed together with the actual raw data. Then LST was converted to °C and EVI was converted back to its native range between -1 and +1.

Mean annual rainfall data with 1 km spatial resolution was obtained from the WorldClim–Global Climate Data website (http://www.worldclim.org/).

All above environmental data were then combined with the houshold position data in a GIS database using Manifold System 8.0 Professional Edition (Manifold Net Ltd, Carson City, NV), which was then used to calculate the following parameters.

Distance to the nearest water course, defined as the shortest distance between the participant’s household and the closest river or stream, and distance to the nearest lake were calculated using the household positions and the above referenced data on lakes and water courses. As elaborated further below, all lakes other than Lake Nyasa were too far away (>4 km) from participant's homes to play an important role in their daily water contact activities.

Household positions and number of inhabitants that had been collected during the initial population census were used to calculate population densities by dividing the number of inhabitants within a 1 km buffer around the participant's home by the surface area of this buffer. Similarly, LST, EVI, rainfall and elevation data were averaged for a buffer area of 1000 m radius around each household to characterize the situation in the area where many of the daily activities of participants would be performed. This approach was preferred to using the respective spot values at the household position, because spot data are more prone to random error than averages for a wider area.

### Statistical analyses

Descriptive analyses were performed using Stata statistics software (Release 14. College Station, TX: StataCorp LP). Maps exploring the prevalence were generated using Manifold System 8.0 Professional Edition (Manifold Net Ltd, Carson City, NV). Geostatistical models were estimated in R version 3.5.0 [30] using the package “gamm4” [31].

Since the majority of *S*. *haematobium* infections were of light intensity and our primary interest was to identify factors related to the presence/absence of infection, we used a binary (no/yes) infection outcome for most of our models. Mixed effects logistic regression with random effects for the study sites and households was used to report odds ratios, which is a suitable method for analysis of clustered cross-sectional data [32].

The following variable transformations were applied to enhance interpretability of results: The reported odds ratios (ORs) correspond to an increase of 1000 persons/km^2^ for the population density, 100 m for elevation, 100 mm for annual rainfall and 0.1 units for EVI. For EVI, LST day and LST night we considered minimum, average and maximum values and included the representation of the respective factor leading to the model with the lowest Akaike Information Criterion (AIC). Age of the participants was stratified into five categories based on the typical *S*. *haematobium* infection patterns over age. Distance to lake was also calculated from remotely sensed data, which however only feature large waterbodies, but do not include small ponds and pools, which also play an important role in schistosomiasis transmission. This variable was stratified into four categories based on the assumed relevance with regard to water contact activities; distances of 4 km and more were deemed too far away for daily lake-water contact. Since only participants in Kyela lived closer than 4 km to a lake (in this case Lake Nyasa), the three lower strata of this variable only include part of the population of Kyela site. All other participants lived more than 4 km away from any lake, including Lake Nyasa. In addition to HIV positive and negative participants, the participants with missing or indecisive HIV test results (371 in total) were included as an additional “no information” stratum into the analysis. A total of 30 observations were discarded due to missing values regarding *S*. *haematobium* infection status or other information, resulting in complete data from 17,280 participants.

First, univariable mixed effects logistic regression with study site and household as random effects was performed to estimate odds ratios of *S*. *haematobium* infection with their 95% confidence intervals for each of the covariates of interest. The random effects were included to account for within-household and within-site clustering of infection.

For our initial multivariable “base” model we included the individual factors age, sex, SES and HIV status (see S1 Table). These variables were included as potential confounders based on their potential relevance for schistosomiasis infection and were left in the model, regardless of their association with *S*. *haematobium* infection. HIV infection was included not only as a potential confounder, but also because we wanted to explore it's relationship with schistosomiasis, since results in the literature regarding this are conflicting. Then, step by step, we included each univariably assessed covariate and left it in the model if the model’s AIC decreased, to identify the most parsimonious model. For more details on the variable selection see S1 Text.

Since environmental data is prone to be highly correlated, we checked for potentially collinear variables by calculating the variance inflation factor (VIF) at each multivariable analysis step. A VIF>10 is mentioned in the literature as an indicator for serious collinearity [33, 34], thus variables with VIF> = 10 were not simultaneously entered into the multivariable models.

Spatial autocorrelation in general refers to situations where nearby observations are more similar than observations further away from each other. Spatial autocorrelation is present in our data, if the probability to be infected with *S*. *haematobium* is clustered in space, which means that it depends on the location where the participant lives. To account for this, we employed geostatistical modelling [35]. We estimated mixed generalized additive logistic models and included a spatially correlated effects base (*i*.*e*. a spatial smoother) on the location of the households and additionally adjusted for clustering of infection by including random effects for study site and household level, respectively. Geostatistical models were estimated for all univariable models, for the multivariable model with the lowest AIC and for the full model including all non-collinear variables (for the full model see S2 Table).

The association of HIV status with the number of excreted *S*. *haematobium* eggs was investigated by means of uni- and multi-variable mixed effects negative binomial regression in participants infected with *S*. *haematobium*. The negative binomial model was chosen to account for the overdispersion present in the data, since the variance of the egg counts was much larger than the mean [36]. We used the egg counts (number of eggs per centiliter of urine) as outcome and HIV status as the main predictor with random effects for study site and household and additionally adjusted for age, sex and SES.

## Results

### Descriptive statistics

**Fig 3:**
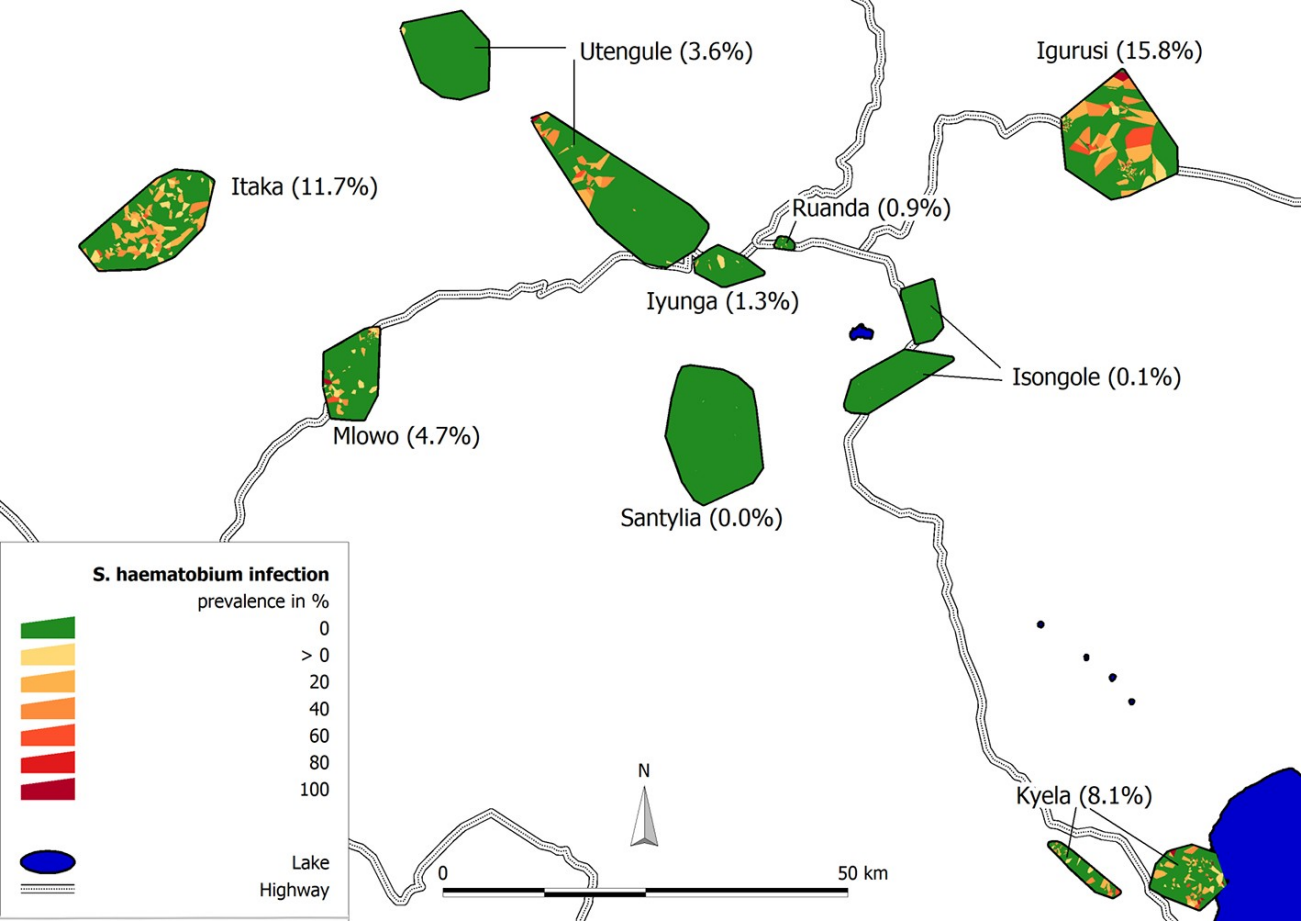
Prevalence of *S*. *haematobium* infection in the nine EMINI study sites in Mbeya region, Tanzania. Households with at least one infected person are represented by yellow to red Voronoi polygons, households without infection are shown in green. (Original work of the authors).

Data from 17280 participants from 4189 households were used for this analysis. The median age of the study population was 17 years (interquartile range (IQR): 9–34 years) and 53% (9194/17280) were females (Table 1). The overall prevalence of *S*. *haematobium* infection in all nine sites was 5.3% (914/17280, 95% confidence interval (CI): 5.0–5.6%), ranging from 0.0 to 15.8% per site (Fig 3 and Table 2). In total the infection occurred in 13.8% of all households (577/4189). Restricting the analyses to school-aged children between 5 and 18 years of age only, an overall prevalence of 9.1% (CI: 8.4–9.8%) was found with site-specific prevalences varying between 0.0 and 29.9% (Table 2). For children under 5 years of age the prevalences were low (see S3 Table). Infections were mostly of light intensity (815/914, 89.2%), heavy infections were found in 99 of the 914 infected study participants (10.8%). Prevalences for the following survey rounds are reported in the S4 Table.

**Table 1:** Characteristics of study participants and environmental conditions at their places of residence.

Variable (Unit)		All sites	Igurusi	Santylia
**Number of participants**	N	17280	1947	2003
**Sex female**	%	53	52	55
**Age** (years)	Median	17	19	17
	(IQR)	(9–34)	(9–36)	(8–34)
***S*. *haematobium* infection prevalence**	%	5.3	15.8	0
**Infection intensity**:				
No infection (0 eggs/10 ml)	%	94.7	84.2	100
Light infection (1–49 eggs/10 ml)	%	4.7	12.7	0
Heavy infection (≥50 eggs/10 ml)	%	0.6	3.1	0
**HIV infection**				
No	%	90.9	88.7	91.8
Yes	%	7.0	6.7	5.6
No information	%	2.1	4.6	2.6
**SES score**	Median	-0.10	-0.09	-0.44
	(IQR)	(-0.57–0.46)	(-0.54–0.48)	(-0.78 –-0.07)
**Population density** (persons/km^2^)	Median	442	1739	205
	(IQR)	(221–2122)	(732–2279)	(132–285)
**Household with latrine**				
No	%	2.4	1.0	1.7
Yes	%	97.6	99.0	98.3
**Elevation** (m)	Median	1579	1193	2025
	(IQR)	(1216–1728)	(1156–1205)	(1982–2066)
**LST Day** (°C)	Median	33.2	34.6	29.9
	(IQR)	(30.4–33.9)	(34.2–36.2)	(29.3–30.8)
**LST Night** (°C)	Median	14.0	16.2	11.7
	(IQR)	(11.8–16.0)	(16.0–16.5)	(10.9–12.0)
**Rainfall** (mm)	Median	1254	1234	1527
	(IQR)	(1156–1605)	(1229–1241)	(1448–1612)
**Slope** (°)	Median	2.3	1.2	6.6
	(IQR)	(1.3–4.4)	(1.1–1.4)	(6.0–7.3)
**Distance to water course** (in km)	Median	1.1	0.9	1.3
	(IQR)	(0.7–1.7)	(0.5–1.4)	(0.8–2.1)
**EVI minimum**	Median	0.17	0.16	0.20
	(IQR)	(0.15–0.20)	(0.15–0.18)	(0.19–0.21)
Distance to Lake Nyasa* (in km)				
Below 1	%	1.1	0	0
1–2	%	2.2	0	0
2–4	%	2.5	0	0
4 and above	%	94.2	100	100

Results for all study sites combined, for the site with the highest infection prevalence (Igurusi) and the site without infection (Santylia). IQR = interquartile range, SES = socio-economic status, LST = land-surface temperature, EVI = enhanced vegetation index.

*Distances below 4 km only apply to participants from Kyela site

**Table 2:** Pre-treatment *S*. *haematobium* infection prevalences in the nine EMINI study sites in Mbeya Region, Tanzania.

	*S*. *haematobium* (total population)			*S*. *haematobium* (school-aged children)		
Study site	No. of participants (households)	No. of infections	Infection prevalence % (95% CI)	No. of participants	No. of infections	Infection prevalence % (95% CI)
**Igurusi**	1947	307	15.8	695	208	29.9
	(514)		(14.2–17.5)			(26.6–33.4)
**Itaka**	1924	225	11.7	809	171	21.1
	(369)		(10.3–13.2)			(18.5–24.1)
**Kyela**	2261	184	8.1	853	96	11.3
	(534)		(7.1–9.3)			(9.3–13.6)
**Mlowo**	2139	100	4.7	898	82	9.1
	(494)		(3.9–5.7)			(7.4–11.2)
**Utengule**	1722	62	3.6	627	36	5.7
	(454)		(2.8–4.6)			(4.2–7.9)
**Iyunga**	1503	19	1.3	641	10	1.6
	(336)		(0.8–2.0)			(0.8–2.9)
**Ruanda**	1676	15	0.9	669	6	0.9
	(406)		(0.5–1.5)			(0.4–2.0)
**Isongole**	2105	2	0.1	806	1	0.1
	(542)		(0.0–0.4)			(0.0–0.9)
**Santylia**	2003	0	0	730	0	0
	(540)					
**All Sites**	**17280**	**914**	**5.3**	**6728**	**610**	**9.1**
	**(4189)**		**(5.0–5.6)**			**(8.4–9.8)**

Shown are the prevalences in the total population (left) and for school-aged children only (5 to 18 years, right). CI = confidence interval.

### Univariable analysis of potential risk factors

**Fig 4:**
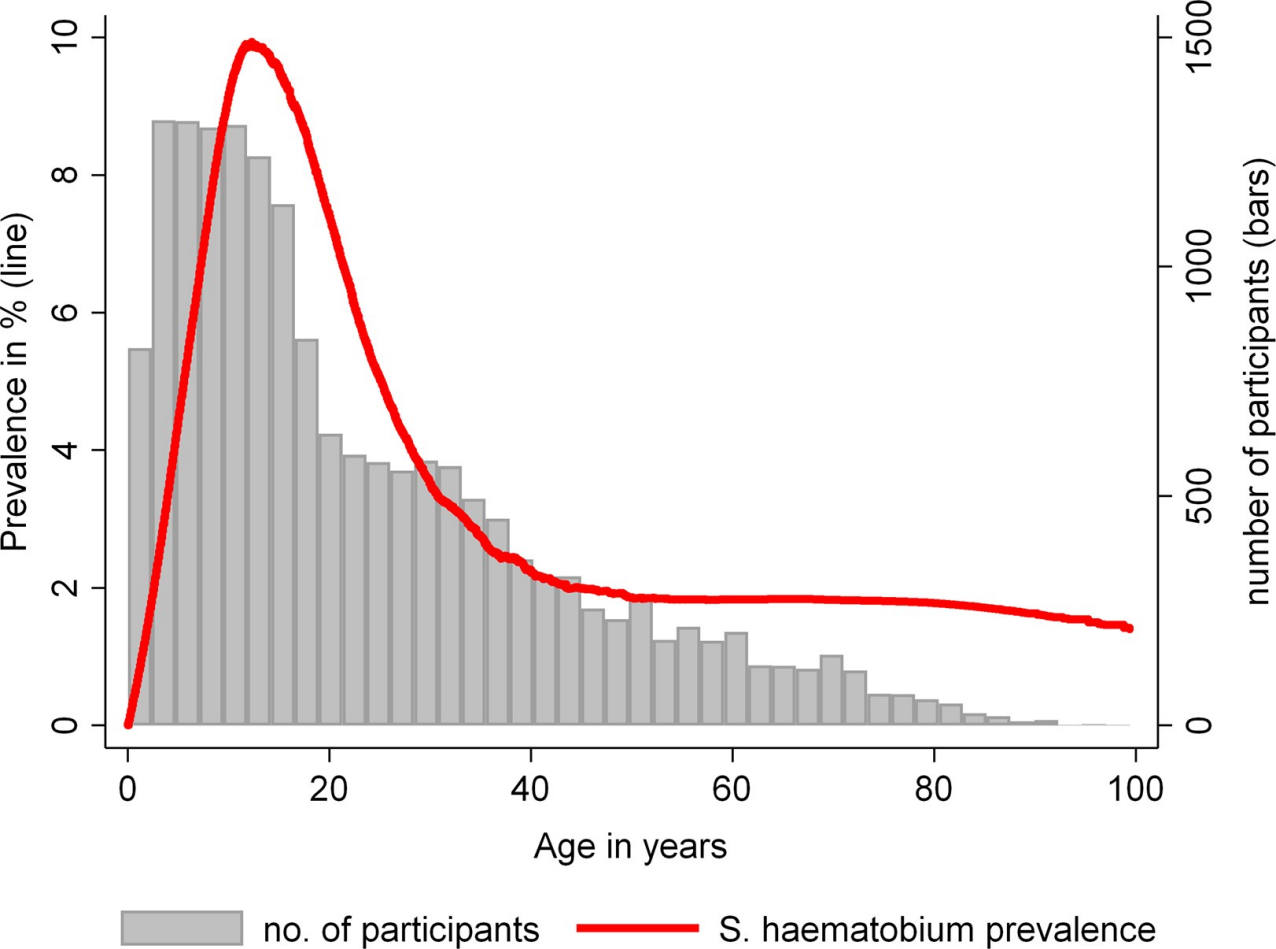
*S*. *haematobium* prevalence by age. The red line shows LOWESS-smoothed *S*. *haematobium* infection prevalence, grey bars indicate the number of participants in each age stratum.

Results of the univariable analysis are shown in Table 3. Using non-spatial modelling we found a statistically significant positive association between *S*. *haematobium* infection and number of persons in the household, land-surface temperature during the night, distance to nearest water course and distance to Lake Nyasa between 2 and 4 km when compared to a distance of 4 km or more. Compared to participants above 35 years of age, younger participants had higher odds of infection. The results were significant for the age groups 5–15, 15–25 and 25–35 years. Children aged 0–5 years were not significantly different from the reference group in terms of *S*. *haematobium* infection status. The age structure of our cohort and the infection prevalence over age are shown in Fig 4.

**Table 3:** Univariable association of socio-demographic and environmental factors with *S*. *haematobium* infection.

All study sites (N = 17280)			Univariable non-spatial*			Univariable spatial^§^		
Covariate	N	% pos.	OR	95% CI	p value	OR	95% CI	p value
**Sex**								
Female*	9194	4.95	1.00	-	-	1.00	-	-
Male	8086	5.68	1.11	0.95–1.29	0.1865	1.09	0.94 – 1.27	0.2300
**Age** (years)								
below 5	2233	2.15	1.16	0.77–1.74	0.4741	1.19	0.79 – 1.79	0.4105
5–15	5534	8.98	8.04	6.05–10.70	**<0.0001**	8.00	6.04 – 10.60	**<0.0001**
15–25	3063	7.61	5.97	4.40–8.10	**<0.0001**	5.96	4.41 – 8.06	**<0.0001**
25–35	2336	2.65	1.60	1.09–2.35	**0.0161**	1.63	1.11 – 2.39	**0.0128**
35 and above*	4114	1.80	1.00	-	**-**	1.00	-	-
**HIV infection**								
No*	15700	5.57	1.00	-	-	1.00	-	-
Yes	1209	2.07	0.32	0.21–0.51	**<0.0001**	0.32	0.21 – 0.51	**<0.0001**
No information	371	4.04	0.49	0.27–0.88	**0.0176**	0.51	0.29 – 0.91	**0.0219**
**SES score** (per 1 unit)			0.92	0.82–1.03	0.1483	0.99	0.88 – 1.10	0.8300
**Number of persons in household** (per person)			1.05	1.02–1.09	**0.0024**	1.05	1.02 – 1.08	**0.0012**
**Population density** (per 1000 persons/km^2^)			1.02	0.93–1.13	0.6510	1.20	1.06 – 1.36	**0.0045**
**Household with latrine**								
No*	419	8.35	1.00	-	-	1.00	-	-
Yes	16861	5.21	0.63	0.37–1.09	0.0970	0.64	0.39 – 1.04	0.0709
**Elevation** (per 100 m)			0.71	0.63–0.80	**<0.0001**	0.72	0.51 – 1.00	0.0526
**LST day** (per 1°C)			1.04	0.96–1.13	0.2950	1.30	1.11 – 1.53	**0.0014**
**LST night** (per 1°C)			1.34	1.16–1.56	**0.0001**	1.27	0.92 – 1.75	0.1468
**Rainfall** (per 100 mm)			0.99	0.89–1.10	0.8977	0.88	0.65 – 1.19	0.4030
**Slope** (per 1°)			0.95	0.88–1.04	0.2794	0.88	0.80 – 0.97	**0.0091**
**Distance to water course** (in km)			1.29	1.15–1.45	**<0.0001**	1.15	1.02 – 1.30	**0.0207**
**EVI minimum** (per 0.1 units)			0.48	0.31–0.73	**0.0008**	0.19	0.09 – 0.37	**<0.0001**
**EVI average** (per 0.1 units)			0.63	0.41–0.94	**0.0255**	0.34	0.20 – 0.60	**0.0001**
**EVI maximum** (per 0.1 units)			0.93	0.67–1.29	0.6585	0.68	0.46 – 1.00	0.0502
**Distance to Lake Nyasa** (in km)								
below 1	195	9.23	1.63	0.78–3.43	0.1974	3.71	0.78 – 17.60	0.0994
1–2	372	9.14	1.56	0.86–2.83	0.1397	3.26	0.95 – 11.21	0.0611
2–4	428	11.92	2.15	1.25–3.70	**0.0059**	4.01	1.59 – 10.07	**0.0032**
4 and above*	16285	4.98	1.00	-	**-**	1.00	-	-

*Univariable mixed effects logistic regression model with random effects for households and study sites (N = 17,280).

^§^Univariable generalized additive mixed model of the binomial family with spatially correlated effects base on the location of the observation, with additional random effects for households and study sites (N = 17,280). The outcome for both modelling approaches was the binary *S*. *haematobium* infection status. N = number of participants, % pos. = prevalence of *S*. *haematobium* infection for categorical variables, OR = odds ratio, CI = confidence interval, SES = socio-economic status, LST = land-surface temperature, EVI = enhanced vegetation index. The reference category for categorical variables is denoted by the asterisk *.

In the non-spatial models we found statistically significant negative univariable associations of *S*.*haematobium* infection with HIV infection, elevation and EVI.

Incorporating the spatial component into the univariable analyses (Table 3) resulted in the following changes: Population density (*i*.*e*. number of persons per km^2^) and LST day became significant positive predictors of *S*. *haematobium* infection pointing towards higher odds of infection with increasing population and with higher LST during the day. Slope became a significant negative predictor of schistosomiasis infection with steeper slopes corresponding to lower odds of infection. In contrast, LST during the night and elevation turned non-significant when taking the spatial correlation into account.

### Multivariable analysis of potential risk factors

In the multivariable non-spatial and spatial models (Table 4), the effect of age on *S*. *haematobium* infection remained similar to that of the univariable models: compared to participants above 35 years of age, younger participants between 5 and 35 years were more likely to be infected with *S*. *haematobium*. Another significant positive association in both modelling approaches was found for the distance to water course, meaning that the odds of *S*. *haematobium* infection increased with increasing distance from water course. Regarding distance to Lake Nyasa, higher odds of infection were found for short distances to the lake compared to the reference category of 4 km and above for both non-spatial and spatial models, respectively.

**Table 4:** Multivariable association of socio-demographic and environmental factors with *S*. *haematobium* infection.

All study sites (N = 17280)			Multivariable non-spatial*			Multivariable spatial^§^		
Covariate	N	% pos.	OR	95% CI	p value	OR	95% CI	p value
**Sex**								
Female*	9194	4.95	1.00	-	-	1.00		
Male	8086	5.68	1.03	0.87–1.21	0.7588	1.02	0.88 – 1.20	0.7653
**Age** (years)								
below 5	2233	2.15	1.15	0.77–1.74	0.4935	1.12	0.74 – 1.68	0.5932
5–15	5534	8.98	7.82	5.85–10.43	**<0.0001**	7.26	5.50 – 9.60	**<0.0001**
15–25	3063	7.61	5.84	4.30–7.95	**<0.0001**	5.51	4.09 – 7.42	**<0.0001**
25–35	2336	2.65	1.64	1.12–2.40	**0.0119**	1.57	1.07 – 2.29	**0.0213**
35 and above*	4114	1.80	1.00	-	**-**	1.00	-	**-**
**HIV infection**								
No*	15700	5.57	1.00	-	-	1.00	-	**-**
Yes	1209	2.07	0.64	0.40–1.03	0.0637	0.63	0.39 – 1.00	0.0524
No information	371	4.04	0.70	0.36–1.33	0.2752	0.72	0.39 – 1.34	0.3021
**SES score** (per 1 unit)			0.90	0.79–1.02	0.1004	0.89	0.79 – 1.01	0.0631
**Population density** (per 1000 persons/km^2^)			1.08	0.97–1.20	0.1559	1.27	1.10 – 1.46	**0.0008**
**Elevation** (per 100 m)			0.74	0.64–0.84	**<0.0001**	0.69	0.46 – 1.04	0.0750
**EVI minimum** (per 0.1 units)			0.21	0.11–0.39	**<0.0001**	0.15	0.07 – 0.33	**<0.0001**
**Distance to water course** (in km)			1.37	1.20–1.57	**<0.0001**	1.17	1.03 – 1.34	**0.0186**
**Distance to Lake Nyasa** (in km)								
below 1	195	9.23	4.53	1.80–11.38	**0.0013**	4.96	0.91 – 27.08	0.0642
1–2	372	9.14	3.51	1.65–7.47	**0.0011**	4.11	1.06 – 15.94	**0.0413**
2–4	428	11.92	3.33	1.68–6.59	**0.0006**	4.31	1.56 – 11.93	**0.0048**
4 and above*	16285	4.98	1.00	-	**-**	1.00	-	**-**

*Multivariable mixed effects logistic model with random effects for households and study sites (N = 17,280).

^§^Multivariable generalized additive mixed model of binomial family with spatially correlated effects base on the location of the observation, with additional random effects for households and study sites (N = 17,280). The outcome for both modelling approaches was the binary *S*. *haematobium* infection status. N = number of participants, % pos. = prevalence of *S*. *haematobium* infection for categorical variables, OR = prevalence ratio, CI = confidence interval, SES = socio-economic status, EVI = enhanced vegetation index. The reference category for categorical variables is denoted by the asterisk *.

We found a negative association of *S*. *haematobium* infection with minimum EVI for both modelling approaches. For the non-spatial model the odds of infection decreased 4.8 fold (1/0.21) for each 0.1 unit increase in EVI and for the spatial model the decrease of odds of infection was with the factor of 6.7 (1/0.15) for each 0.1 unit increase even higher. Elevation showed non-spatially a highly significant association with *S*. *haematobium* infection (OR = 0.74, CI: 0.64–0.84), which didn’t reach statistical significance after taking the spatial correlation into account (OR = 0.69, CI: 0.46–1.04). In contrast to this, population density became highly significant in the spatial model (non-spatial: OR = 1.08, CI: 0.97–1.20; spatial: OR = 1.27, CI: 1.10–1.46).

Elevation and LST during day and night both had strong univariable associations with *S*. *haematobium* infection but were also strongly collinear, which prevented inclusion into the same multivariable model. Since elevation is more often used in the literature than LST, we decided to include elevation in our models for better comparability. The negative association of HIV with *S*. *haematobium* infection, found in the univariable analysis, was not significant anymore in the multivariable models, although the adjusted odds of *S*. *haematobium* infection in HIV-positive participants were still lower than in HIV-negative participants (non-spatial: OR = 0.64, CI: 0.40–1.03; spatial OR = 0.63, CI: 0.39–1.00). Gender and socio-economic status, which were included in the models as potential confounders, were both not significantly associated with *S*. *haematobium* infection.

### HIV and egg excretion

Among the 914 *S*. *haematobium* infected participants, a total of 25 individuals were co-infected with HIV. The geometric mean egg counts per centiliter of urine were 3.3 (CI: 1.8–6.2) and 4.8 (CI: 4.3–5.4) EPC in HIV positive and HIV negative individuals, respectively. When analyzing the association of egg counts with HIV infection in these 914 participants, the univariable mixed effects negative binomial model showed an egg count ratio of 0.90 (95% CI: 0.69–1.17) for HIV positive compared to HIV negative individuals (see S5 Table). When additionally adjusting for age, sex, and SES, an egg count ratio of 1.68 (95% CI: 0.98–2.90) for HIV positive, compared to HIV negative participants was found, showing a trend towards increased *S*. *haematobium* egg excretion in HIV infected individuals.

## Discussion

Our results show significant associations of individually assessed and remotely sensed factors with *S*. *haematobium* infection in Mbeya Region in Southwestern Tanzania. Using multivariable modelling, we found increased odds of infection in school-age children and young adults, with increasing distance to water course and decreasing distance to Lake Nyasa, whereas higher vegetation cover was associated with lower odds of infection. After including the spatial component into the analyses, elevation was not significant anymore, whereas population density became a significant predictor of schistosomiasis. The overall prevalence was 5.3%, ranging from 0 to 15.8% in the different study sites showing the spatial heterogeneity which is typical for schistosomiasis. These prevalences are in line with spatial predictions made for Tanzania by Brooker et al., where the prevalence predicted for our study region was also low (*i*.*e*. the estimated probability for our study area having an infection prevalence >50% was 0–10%) [37]. Considering only school-age children, we found a higher overall prevalence of 9.1% with site-specific prevalences up to 29.9%.

Male sex did not show a statistically significant association with *S*. *haematobium* infection in our study, even after spatial adjustment. Previous studies report inconsistent associations of sex with *S*. *haematobium* prevalence [15, 38–42]. Women and men can have different water contact behaviour relating to activities such as swimming, fishing, and doing laundry but the frequency and type of water contact also depend on the water sources available in a community.

Age is a significant predictor of *S*. *haematobium* infection. In our study population, schistosomiasis mainly affects children and young adults, which is consistent with the literature [2, 43]. Many other studies are concerned with quantifying *S*. *haematobium* infection in children only, whereas we report results from a population-based study including all age groups and thus show the distribution of *S*. *haematobium* infection in the whole population.

The assessment of HIV as a factor with potential influence on *S*. *haematobium* infection is limited by the fact that both diseases show distinct age patterns with peaks at different ages: As opposed to *S*. *haematobium* infection, which is most prevalent in school age children, the prevalence of HIV peaks at around 35 years of age, when *S*. *haematobium* prevalence has already dropped to low levels. Thus the strong negative association of HIV and *S*. *haematobium* infection in the univariable analysis (OR = 0.40) is at least partly due to the different age peaks that both diseases occur at, although a similar trend towards lower *S*. *haematobium* prevalence in HIV infected participants is also visible in the age-adjusted multivariable model (non-spatial OR = 0.64). We further investigated the relationship between *S*. *haematobium* egg excretion and HIV by means of mixed effects negative binomial regression with egg-counts as the outcome in *S*. *haematobium* infected participants only. The adjusted model showed a trend towards increased *S*. *haematobium* egg excretion in HIV co-infected individuals (S5 Table). This positive association mainly results from the inclusion of age as a covariate into the model, and again is caused by different age/infection patterns of the two diseases.

Results in the literature regarding the interaction between HIV and schistosomiasis are conflicting. Some studies have found an increased risk of HIV transmission in schistosomiasis infected individuals [44–46], which is especially severe for women affected by genital schistosomiasis [8]. However, microscopic diagnosis of schistosomiasis in HIV infected individuals appears to lack sensitivity [47, 48]. Several articles describe significantly [49–52] or non-significantly [53, 54] decreased *S*. *mansoni* and/or *S*. *haematobium* egg excretion in HIV co-infected individuals. Other studies find no association of HIV status with schistosome egg-excretion after adjusting for age [55, 56] or even slightly higher mean egg counts (non-significant) in HIV-positives [57]. Some of these differing findings could be explained by different levels of immune suppression in the populations that were studied [50, 52, 58], and by sex differences regarding the impact HIV has on Schistosoma egg excretion [47].

We found a significant positive univariable association of *S*. *haematobium* infection with the number of household members, but this effect vanished when adjusting for other covariates, thus it was likely caused by confounding, e.g. larger households in an area with high infection prevalence. However, Sady *et al*. found an association between schistosomiasis and presence of other infected family members, which also remained significant in the multivariable analysis [59]. It seems a likely explanation that members of the same household use the same water bodies for their daily activities which leads to household clustering of the infection.

We had expected a significant association between socioeconomic status and *S*. *haematobium* infection since *S*. *haematobium* infection is regarded as a disease of poverty. Our findings were not statistically significant, but nevertheless, the odds of infection decreased with increasing SES. Previous studies have reasoned that those living at poverty level are at an increased risk of schistosomiasis due to a greater exposure to unsafe water, less education about transmission and exposure reduction strategies, and limited access to effective treatment [6, 60, 61]. In our study SES showed the strongest association with *S*. *haematobium* infection in the spatially adjusted models. Another study where SES was calculated in a manner similar to ours, found an association between SES and *S*. *haematobium* infection [62]. One reason for the lack of association might be that the vast majority of participants in the rural study sites do not have access to safe water for bathing, swimming and washing of clothes, independent of SES.

Population density showed a statistically significant positive association with *S*. *haematobium* infection only in the spatially adjusted models, indicating increased tranmsmission in densely populated areas. A similar association was found by Koroma *et al*. using geostatistical modelling [17]. In a study conducted by Nagi *et al*., population density was a significant risk factor for *S*. *mansoni* infection [63].

Living at higher altitude was associated with reduced odds of urinary schistosomiasis, which is also found in other studies [e.g. 20, 64–66]. Higher altitude is associated with steeper slopes and less stagnant water, which in turn leads to less temporary water bodies and to less suitable habitats for the snails. In our study the site with no infected participants was the highest altitude site. High elevation is associated with lower temperatures, too. In the univariable analysis LST night was highly significantly associated with *S*. *haematobium* infection indicating higher odds of infection with increasing night-time temperature. However, after spatial adjustment this effect turned non-significant. This finding is in line with the study by Clements *et al*. [13], where an association between *S*. *haematobium* and minimum LST was found, which turned non-significant, when including the spatial correlation into the model. Schur *et al*. [20] used spatial modelling and found a non-linear relation between *S*. *haematobium* with night LST indicating a positive relation and a decrease in risk at the lowest and the highest values LST.

Regarding LST day in the univariable analysis, we found a positive significant effect only after including the spatial correlation structure. While Clark *et al*. also found a positive association between *S*. *mansoni* and LST using geostatistical models [67], negative associations were found in other studies [20, 68].

Slope of the terrain showed negative odds of infection after spatial adjustment of the univariable model, indicating that steeper slopes lead to decreased *S*. *haematobium* infection. Kulinkina *et al*. also found a negative association of slope and *S*.*haematobium* infection [69]. Probable reasons for this negative association of slope with infection are the same as those discussed above for increasing altitude.

As opposed to other studies, we found no stronger associations of rainfall with *S*. *haematobium* infection [20, 68]. Such an association was also found by Clements et al. [13], it however diminished when incorporating the spatial correlation into the analysis. Potential explanations why rainfall did not appear to play an important role in our study, might be a lack of variation in rainfall in our relatively small study area, the low spatial resolution of the rainfall data and the lack of seasonal resolution both for the rainfall and for the schistosomiasis data.

Considering that *S*. *haematobium* infection requires contact with contaminated water, it was surprising to find an increased prevalence of infection with increasing distance to the nearest water course, i.e. stream or river. Water velocity is an important factor in the transmission of schistosomiasis since the survival of the intermediate host snails can be impacted in fast-flowing water [70]. Even if Rabone *et al*. [71] found a negative association between water speed and *Bulinus spp*. abundance, this does not rule out that the snails may tolerate medium flow speeds for short periods of time. *Bulinus spp*. generally seem to prefer low flow environments [72]. Furthermore, shallow water bodies and still waters are conducive to the growth of algae and aquatic plants on which the snail intermediate hosts feed [73]. Lakes, ponds and pools thus provide the conditions favored by both the snail intermediate host and the free-swimming forms of the schistosome. People living further away from a stream likely more often use ponds or pools to do their washing, swimming and fishing, and thus increase their risk of infection. This is also supported by our finding that *S*. *haematobium* prevalence increased with decreasing distance to Lake Nyasa in the one site that is situated close to the lake. In the literature, one study found an increased prevalence of schistosomiasis in children living close to streams, springs, pools or ponds [59], whereas in another study, the distance to river was not associated with *S*. *haematobium* infection [62]. For our study, information regarding smaller stagnant waterbodies would have been interesting, but unfortunately our remotely sensed data only contained information regarding the few larger lakes in the area. We also have no malacological information regarding the presence of suitable host snails in our study area, but at least for Lake Nyasa, *Biomphalaria pfeifferi* (intermediate host snails for *S*. *mansoni*) have been found in the southern parts of the lake [74].

We found decreased odds of *S*. *haematobium* infection with increasing amount of vegetation, as measured by the EVI. Sturrock *et al*. [75] also observed significantly decreased odds of *S*. *haematobium* infection with increasing EVI in their model without spatial component, which however vanished after including the spatial component into the analyses. Some studies have found a significant positive association between *S*. *haematobium* and/or *S*.*mansoni* and the Normalized Difference Vegetation Index (NDVI), which is very similar to EVI [19, 67, 76], whereas in other studies NDVI was not associated with schistosomiasis in the preliminary analyses and thus excluded from further analyses [77, 78]. Another study observed a non-linear association between schistosomiasis and NDVI [20]. However, EVI characterizes the land vegetation cover rather than aquatic vegetation, which is more relevant to the intermediate host snails. A recent study by Wood *et al*. [79] found tight associations between certain kinds of aquatic vegetation and the intermediate host snails, which further hints towards aquatic vegetation being the key for schistosomiasis transmission. Another possible explanation of the reversed association between schistosomiasis and EVI is related to population density. Lower EVI also indicates higher land-clearing and higher population density. Indeed, the two sites with the highest population density had the lowest EVI.

Our study has several limitations: First, data for this study came from a general survey and data collection was not specifically targeted to investigate risk factors of *S*. *haematobium* infection. Our study would have benefited from more focused questions regarding water contact activities, exposure and occupation. Although some of the environmental factors considered here were strongly related to schistosomiasis, they probably do not fully explain the spatial pattern of *S*. *haematobium* infection in the study area, since we neither have information regarding the occurrence of suitable snail intermediate hosts, nor data regarding smaller stagnant or temporary waterbodies, which both would be important to get a better picture of transmission potential in an area. While such data collection might be feasible for small-scale studies, it is not easily available across larger spatial scales. Two recent studies have shown that intermediate host snail populations are very patchy in time and space [71, 79] and thus targeting them for control campaigns would require effort exceeding what is feasible. However, Wood *et al*. identified environmental proxies for the snails which were shown to better predict schistosomiasis infection risk than the actual snail abundance data. The area of snail-suitable habitat within a water contact site and the total area of the water contact site were identified as such environmental proxies [79]. Since we are lacking data on small ponds and water contact sites, such analyses are left for future studies.

Due to collinearity with elevation, we couldn’t include LST into multivariable models and thus cannot evaluate its impact after adjusting for other important factors. We collected two urine samples (one morning and one spot sample between 10 am and 2 pm for a maximized egg output [80]) but may still have missed some light infections: especially chronically infected adults pass fewer eggs in the urine and where lesions and fibrous tissue have already developed, more eggs are trapped within the body [81].

Despite these limitations, our study also has a couple of unique strengths. The study covers a medium sized area with a large and representative study population of more than 17,000 participants, for which we have individual data regarding *S*. *haematobium* infection, place of residence and individual risk factors. In contrast, most other studies cited here either have individual data on relatively small study populations from few communities, or they cover entire provinces or countries, and use school prevalences instead of individual outcome data, which means that the population above school age is not considered, and that only pooled data on outcomes and risk factors can be analysed. Regarding some of the different findings of our study compared to others, and also between other studies, one should keep in mind that "scale matters" [82], i.e. that the above mentioned differences in scale between studies might also explain some of the different findings.

### Conclusions

*S*. *haematobium* infection investigated in this study in Mbeya region of Southwestern Tanzania revealed highly focal infection with prevalences between 0 and 16% in the different study sites.

Our study did not find any *S*. *haematobium* infection in the site with the highest altitude. The inverse relationship between elevation and *S*. *haematobium* infection indicates that lower altitude sites are at higher risk of schistosomiasis. We also found a strong negative association of EVI with schistosomiasis and a strongly elevated infection risk in children. Elevation and vegetation density can easily be obtained from remote sensing data that are available free of charge in the public domain, whereas age data are not easy to obtain. Proximity to Lake Nyasa was also associated with increased odds of infection. The positive association of distance to flowing water might suggest that smaller temporary water bodies potentially housing infected intermediate host snails are preferred when the river is too far away for regular water contacts. Population density was another important risk factor for the infection. Regarding public health implications, people living at low altitude and school-aged children living in crowded areas might be considered as the persons at higher risk for *S*. *haematobium* infection. These findings from our large population-based study of more than 17,000 participants and the reported pre-treatment prevalences could help to evaluate and improve ongoing and future control activities in the region, in Tanzania and elsewhere.

## Supporting information

S1 Checklist STROBE Checklist. (DOC)

S1 Table Multivariable association of socio-demographic factors only with *S*. *haematobium* infection. Results of multivariable mixed effects logistic regression with site and household as random effects and with binary S. haematobium infection status as the outcome (N = 17,280). *Multivariable mixed effects logistic model with random effects for households and study sites. ^§^Multivariable generalized additive mixed model of binomial family with spatially correlated effects base on the location of the observation, with additional random effects on households and study sites. OR = odds ratio, CI = confidence interval, SES = socio-economic status. The reference category for stratified variables is denoted by the asterisk *.
(DOCX)

S2 Table Full model: Association of socio-demographic and environmental factors with S. haematobium infection. *Multivariable mixed effects logistic model with random effects for households and study sites. ^§^Multivariable generalized additive mixed model of binomial family with spatially correlated effects base on the location of the observation, with additional random effects for households and study sites.
(DOCX)

S3 Table *S*. *haematobium* infection prevalences in children below 5 years of age. CI: confidence interval. (DOCX)

S4 Table Prevalence of *S*. *haematobium* infection over all survey rounds of the EMINI study. CI: confidence interval.
(DOCX)

S5 Table Association of HIV status with *S*. *haematobium* infection intensity. Results of multivariable mixed effects negative binomial regression with site and household as random effects and with *S*. *haematobium* egg counts as the outcome, adjusted for socio-demographic factors. Only includes *S*. *haematobium* infected participants (N = 914). ECR = egg count ratio, CI = confidence interval, SES = socio-economic status. The reference category for stratified variables is denoted by the asterisk *.
(DOCX)

S1 Text Details on variable selection. (DOCX)
