# Supplementary material for: Schistosoma haematobium infection and environmental factors in Southwestern Tanzania: A cross-sectional, population-based study
Source: PLoS Negl Trop Dis. 2020 Aug 24;14(8):e0008508. doi: 10.1371/journal.pntd.0008508 (PMC7446842; doi:10.1371/journal.pntd.0008508)
Supplement: S1 Checklist — (DOC) [file pntd.0008508.s001.doc]

STROBE Statement—Checklist of items that should be included in reports of ***cross-sectional studies*** Manz *et al.*, 2020

|  | Item No | Recommendation | Relevant text from manuscript |  |  |
| --- | --- | --- | --- | --- | --- |
| **Title and abstract** | 1 | (*a*) Indicate the study’s design with a commonly used term in the title or the abstract | *Schistosoma haematobium* infection and environmental factors in Southwestern Tanzania, a cross-sectional, population-based study **[Title]** |  |  |
|  |  | (*b*) Provide in the abstract an informative and balanced summary of what was done and what was found | Data regarding socio-demographic status and *S. haematobium* infection were obtained between May 2006 and May 2007 from 17280 participants (53% females, median age = 17 years) in the Mbeya Region, Tanzania. Combined with remotely sensed environmental data (vegetation cover, altitude, rainfall etc.) this data was analyzed to identify environmental and socio-demographic factors associated with *S. haematobium* infection, using mixed effects logistic regression and geostatistical modelling. The overall prevalence of *S. haematobium* infection was 5.3% (95% confidence interval (CI): 5.0-5.6%). Multivariable analysis revealed increased odds of infection for school-aged children (5-15 years, odds ratio (OR)=7.8, CI: 5.9-10.4) and the age groups 15-25 and 25-35 years (15-25 years: OR=5.8, CI: 4.3-8.0, 25-35 years: OR=1.6, CI: 1.1-2.4) compared to persons above 35 years of age, for increasing distance to water courses (OR=1.4, CI: 1.2-1.6 per km) and for proximity to Lake Nyasa (<1 km, OR=4.5, CI: 1.8-11.4; 1-2 km, OR=3.5, CI: 1.7-7.5; 2-4 km; OR=3.3, CI: 1.7-6.6), when compared to distances >4 km. Odds of infection decreased with higher altitude (OR=0.7, CI: 0.6-0.8 per 100 m increase) and with increasing enhanced vegetation index EVI (OR=0.2, CI: 0.1-0.4 per 0.1 units). When additionally adjusting for spatial correlation population density became a significant predictor of schistosomiasis infection (OR=1.3, CI: 1.1-1.5 per 1000 persons/km²) and altitude turned non-significant. **[Abstract (Methodology/Principal findings)]**  Our spatially heterogeneous results show that despite low overall prevalence, some of the study sites suffer from a considerable burden of *S. haematobium* infection, which is related to various socio-demographic and environmental factors. Our results could help to design more effective control strategies in the future, especially targeting school-aged children living in low altitude sites and/or crowded areas as the persons at highest need for preventive chemotherapy. **[Abstract (Conclusions/Significance)]** |  |  |
| Introduction | | |  |  |  |
| Background/rationale | 2 | Explain the scientific background and rationale for the investigation being reported | Trematodes of the genus *Schistosoma* are among the most common infectious agents of humans. **[Introduction]**  *S. haematobium*, the agent causing urinary schistosomiasis, only occurs in Africa and the Middle East, where it is the most common schistosome species [3, 4]. Consequences of urinary schistosomiasis include hematuria, dysuria, bladder scarring, chronic urinary tract infection and possibly bladder cancer [5, 6]. Environmental conditions that are permissive to the development of intermediate host snails are important factors for schistosomiasis transmission in endemic regions. Advances in remote sensing (RS) and geographic information systems (GIS) have enabled researchers to explore these environmental and climatic factors in greater depth. Risk mapping, with the aid of RS and GIS applications, is suited to the study of schistosomiasis as the infectious agents and their snail hosts are sensitive to environmental conditions [10, 11]. **[Introduction]** |  |  |
| Objectives | 3 | State specific objectives, including any prespecified hypotheses | The aim of this study was to report pre-treatment prevalences of *S. haematobium* infection and to identify individual factors and local environmental conditions that might influence *S. haematobium* transmission in Mbeya Region in Southwestern Tanzania. **[End of Introduction section]** |  |  |
| Methods | | |  |  |  |
| Study design | 4 | Present key elements of study design early in the paper | Please see **Methods -> Study area and epidemiological data collection** and text below. |  |  |
| Setting | 5 | Describe the setting, locations, and relevant dates, including periods of recruitment, exposure, follow-up, and data collection | The study area is located in the Mbeya Region in Southwestern Tanzania…  Prior the start of the EMINI study a census covering more than 42,000 households was carried out in the study region. The study area and the nine study sites were preselected to cover a wide variety of economic and environmental conditions. After the census of all households in each site was completed, 10% of the households within these sites were chosen as a geographically stratified random sample to participate in the study. Household positions were determined using handheld GPS devices. Each household was visited annually starting in 2006. During the survey visits we collected blood and urine samples to test for HIV and schistosomiasis, and performed structured interviews in the local language (Kiswahili). An additional stool sample collection for exploring soil-transmitted helminth infections started at the third annual visit from 2008 onwards in 50% of the households. For this analysis we used data from the first annual survey which was conducted between May 2006 and May 2007. **[Methods (Study area and epidemiological data collection)]**  Please see **Methods -> Ecological data** for information on how the ecological data was collected and processed. |  |  |
| Participants | 6 | (*a*) Give the eligibility criteria, and the sources and methods of selection of participants | Prior the start of the EMINI study a census covering more than 42,000 households was carried out in the study region. The study area and the nine study sites were preselected to cover a wide variety of economic and environmental conditions. After the census of all households in each site was completed, 10% of the households within these sites were chosen as a geographically stratified random sample to participate in the study.  **[Methods (Study area and epidemiological data collection)]** |  |  |
| Variables | 7 | Clearly define all outcomes, exposures, predictors, potential confounders, and effect modifiers. Give diagnostic criteria, if applicable | Outcomes:  All participants with urine samples with at least one *S. haematobium* egg were regarded as infected. *S. haematobium* infection intensity was recorded in eggs per centiliter (EPC) and classified into no (0 EPC), light intensity (<50 EPC) and heavy intensity infections (≥50 EPC) according to World Health Organization guidelines [15]. **[Methods (Study area and epidemiological data collection)]**  Since the majority of *S. haematobium* infections were of light intensity and our primary interest was to identify factors related to the presence/absence of the infection, we used a binary infection outcome (no/yes) for most of our models. **[Methods (Statistical analyses)]**  Predictors:  The following variable transformations were applied to enhance interpretability of results: The reported odds ratios (ORs) correspond to an increase of 1000 persons/km2 for the population density, 100 m for elevation, 100 mm for annual rainfall and 0.1 units for EVI. For EVI, LST day and LST night we considered minimal, averaged and maximal values and included the form of the variable leading to the model with the lowest Akaike Information Criterion (AIC). Age of the participants was stratified into five categories based on the typical S. haematobium infection patterns over age. Distance to lake was also calculated from remotely sensed data, which however only feature large waterbodies, but do not include small ponds and pools, which also play an important role in schistosomiasis transmission. This variable was stratified into four categories based on the assumed relevance with regard to water contact activities; distances of 4 km and more were deemed too far away for daily lake-water contact. Since only participants in Kyela lived closer than 4 km to a lake (in this case Lake Nyasa), the three lower strata of this variable only include part of the population of Kyela site. All other participants lived more than 4 km away from any lake, including Lake Nyasa. In addition to HIV positive and negative participants, the participants with missing or indecisive HIV test results (371 in total) were included as an additional “no information” stratum into the analysis.  **[Methods (Statistical analyses)]**  **S1 File**  Confounding:  For our initial multivariable “base” model we included the individual factors age, sex, SES and HIV status (see Supplementary S1 Table). These variables were included as potential confounders based on their relevance to schistosomiasis and were left in the model, regardless of their association with S. haematobium infection. Especially we wanted to explore the relationship between schistosomiasis and HIV infection, since results in the literature regarding this are conflicting. Then, step by step, we included each univariably assessed covariate and left it in the model if the model’s AIC decreased, to identify the most parsimonious model. |  |  |
| Data sources/ measurement | 8* | For each variable of interest, give sources of data and details of methods of assessment (measurement). Describe comparability of assessment methods if there is more than one group | During the annual survey visits we collected blood and urine samples to test for HIV and schistosomiasis, and performed structured interviews in the local language (Kiswahili). **[Methods (Study area and epidemiological data collection)]**  Household positions and number of inhabitants that had been collected during the initial population census were used to calculate population densities. **[Methods (Ecological data)]**  For details about enrironmental data please see **Methods -> Ecological data)**  For calculation of the socio-econimic status please see **Methods -> Study area and epidemiological data collection).** |  |  |
| Bias | 9 | Describe any efforts to address potential sources of bias | For our initial multivariable “base” model we included the individual factors age, sex, SES and HIV status (see Supplementary S1 Table). These variables were included as potential confounders based on their relevance to schistosomiasis and were left in the model, regardless of their association with S. haematobium infection. Especially we wanted to explore the relationship between schistosomiasis and HIV infection, since results in the literature regarding this are conflicting. **[Methods (Statistical analyses)]** |  |  |
| Study size | 10 | Explain how the study size was arrived at | Prior the start of the EMINI study a census covering more than 42,000 households was carried out in the study region. The study area and the nine study sites were preselected to cover a wide variety of economic and environmental conditions. After the census of all households in each site was completed, 10% of the households within these sites were chosen as a geographically stratified random sample to participate in the study. **[Methods (Study area and epidemiological data collection)]**  A total of 30 observations were discarded due to missing values regarding S. haematobium infection status or other information, resulting in complete data from 17,280 participants. **[Methods (Statistical analyses)]** |  |  |
| Quantitative variables | 11 | Explain how quantitative variables were handled in the analyses. If applicable, describe which groupings were chosen and why | The population density, LST, EVI, rainfall and elevation data were averaged for a buffer area of 1000 m radius around each household to characterize the situation around the household. **[Methods (Ecological data)]**  The following variable transformations were applied to enhance interpretability of results: The reported odds ratios (ORs) correspond to an increase of 1000 persons/km2 for the population density, 100 m for elevation, 100 mm for annual rainfall and 0.1 units for EVI. For EVI, LST day and LST night we considered minimal, averaged and maximal values and included the form of the variable leading to the model with the lowest Akaike Information Criterion (AIC). Age of the participants was stratified into five categories based on the typical S. haematobium infection patterns over age. Distance to lake was also calculated from remotely sensed data, which however only feature large waterbodies, but do not include small ponds and pools, which also play an important role in schistosomiasis transmission. This variable was stratified into four categories based on the assumed relevance with regard to water contact activities; distances of 4 km and more were deemed too far away for daily lake-water contact. Since only participants in Kyela lived closer than 4 km to a lake (in this case Lake Nyasa), the three lower strata of this variable only include part of the population of Kyela site. All other participants lived more than 4 km away from any lake, including Lake Nyasa. In addition to HIV positive and negative participants, the participants with missing or indecisive HIV test results (371 in total) were included as an additional “no information” stratum into the analysis. **[Methods (Statistical analyses)]** |  |  |
| Statistical methods | 12 | (*a*) Describe all statistical methods, including those used to control for confounding | Mixed effects logistic regression with random effects at the study sites and households was used to report odds ratios, which is a suitable method for analysis of clustered cross-sectional data.  First, univariable mixed effects logistic regression with study site and household as random effects was performed to estimate odds ratios of S. haematobium infection with their 95% confidence intervals for each of the covariates of interest. The random effects were included to account for within-household and within-site clustering of infection.  For our initial multivariable “base” model we included the individual factors age, sex, SES and HIV status (see Supplementary S1 Table). These variables were included as potential confounders based on their relevance to schistosomiasis and were left in the model, regardless of their association with S. haematobium infection. Especially we wanted to explore the relationship between schistosomiasis and HIV infection, since results in the literature regarding this are conflicting. Then, step by step, we included each univariably assessed covariate and left it in the model if the model’s AIC decreased, to identify the most parsimonious model.  Since environmental data is prone to be highly correlated, we checked for potentially collinear variables by calculating the variance inflation factor (VIF) at each multivariable analysis step.  To account for the spatial autocorrelation geostatistical modelling was employed. More detailed, mixed generalized additive logistic models were estimated where we included a spatially correlated effects base (i.e. spatial smoother) on the location of the households and additionally adjusted for infection clustering by including random effects at study site and household level, respectively. Geostatistical models were estimated for all univariable models, for the multivariable model with the lowest AIC and for the full model including all non-collinear variables. **[Methods (Statistical analyses)]** |  |  |
|  |  | (*b*) Describe any methods used to examine subgroups and interactions | The association of HIV status with the number of excreted *S. haematobium* eggs was investigated by means of uni- and multi-variable mixed effects negative binomial regression in participants infected with *S. haematobium.* We used the untransformed egg counts as outcome and HIV status as the main predictor with random effects for study site and household. For the multivariable analysis we also included age, sex and SES as potential confounders. **[Methods (Statistical analyses)]** |  |  |
|  |  | (*c*) Explain how missing data were addressed | In addition to HIV positive and negative participants, the participants with missing or indecisive HIV test results (371 in total) were included as an additional “no information” stratum into the analysis. A total of 30 observations were discarded due to missing values regarding *S. haematobium* infection status or other information, resulting in complete data from 17,280 participants. **[Methods (Statistical analyses)]** |  |  |
|  |  | (*d*) If applicable, describe analytical methods taking account of sampling strategy | NA |  |  |
|  |  | (*e*) Describe any sensitivity analyses | To account for the spatial autocorrelation geostatistical modelling was employed. More detailed, mixed generalized additive logistic models were estimated where we included a spatially correlated effects base (i.e. spatial smoother) on the location of the households and additionally adjusted for infection clustering by including random effects at study site and household level, respectively. Geostatistical models were estimated for all univariable models, for the multivariable model with the lowest AIC and for the full model including all non-collinear variables. **[Methods (Statistical analyses)]** |  |  |
| Results | | |  |  |  |
| Participants | 13* | (a) Report numbers of individuals at each stage of study—eg numbers potentially eligible, examined for eligibility, confirmed eligible, included in the study, completing follow-up, and analysed | Prior the start of the EMINI study a census covering more than 42,000 households was carried out in the study region…. After the census of all households in each site was completed, 10% of the households within these sites were chosen as a geographically stratified random sample to participate in the study.**[Methods (Study area and epidemiological data collection)]**  A total of 30 observations were discarded due to missing values regarding *S. haematobium* infection status or other information, resulting in complete data from 17,280 participants. **[Methods (Statistical analyses)]** |  |  |
|  |  | (b) Give reasons for non-participation at each stage |  |  |  |
|  |  | (c) Consider use of a flow diagram |  |  |  |
| Descriptive data | 14* | (a) Give characteristics of study participants (eg demographic, clinical, social) and information on exposures and potential confounders | See **Results -> Descriptive Statistics** |  |  |
|  |  | (b) Indicate number of participants with missing data for each variable of interest | A total of 30 observations were discarded due to missing values regarding *S. haematobium* infection status or other information, resulting in complete data from 17,280 participants.  In addition to HIV positive and negative participants, the participants with missing or indecisive HIV test results (371 in total) were included as an additional “no information” stratum into the analysis. **[Methods (Statistical analyses)]** |  |  |
| Outcome data | 15* | Report numbers of outcome events or summary measures | The overall prevalence of *S. haematobium* infection in all nine sites was 5.3% (914/17280, 95% confidence interval (CI): 5.0 - 5.6%), ranging from 0.0 to 15.8% per site (Fig 3., Table 2). **[Results (Descriptive statistics)]**  See Fig.3 for graphical results and Table 2 for tabulated site-specific results. |  |  |
| Main results | 16 | (*a*) Give unadjusted estimates and, if applicable, confounder-adjusted estimates and their precision (eg, 95% confidence interval). Make clear which confounders were adjusted for and why they were included | For unadjusted estimates see Table 3, for adjusted estimates Table 4. |  |  |
|  |  | (*b*) Report category boundaries when continuous variables were categorized | Age categories (Table 3 and 4): Below 5 years, 5-15 years, 15-25 years, 25-35 years, 35 and above. Distance to Lake Nyasa (Table 3 and 4): below 1 km, 1-2 km, 2-4 km, 4 km and above. |  |  |
|  |  | (*c*) If relevant, consider translating estimates of relative risk into absolute risk for a meaningful time period | NA |  |  |
| Other analyses | 17 | Report other analyses done—eg analyses of subgroups and interactions, and sensitivity analyses | When analyzing the association of egg counts with HIV infection in these 914 participants, the univariable mixed effects negative binomial model showed an egg count ratio of 0.90 (95% CI: 0.69 – 1.17) for HIV positive individuals compared to HIV negative individuals (see Supplementary Table S2). When additionally adjusting for age, sex, and SES, an egg count ratio of 1.68 (95% CI: 0.98 – 2.90) for HIV positive, compared to HIV negative participants was found, showing a trend towards increased *S. haematobium* egg excretion in HIV infected individuals. **[Results (HIV and egg excretion)]** |  |  |
| Discussion | | |  |  |  |
| Key results | 18 | Summarise key results with reference to study objectives | Using multivariable modelling, we found increased odds of infection in school-age children and young adults, with increasing distance to water course and decreasing distance to Lake Nyasa, whereas higher vegetation cover was associated with lower odds of infection. After including the spatial component into the analyses elevation was not significant anymore, however, population density became significant predictor of schistosomiasis. The overall prevalence was 5.3%, ranging from 0 to 15.8% in the different study sites showing the spatial heterogeneity typical for schistosomiasis. **[Discussion, paragraph 1]** |  |  |
| Limitations | 19 | Discuss limitations of the study, taking into account sources of potential bias or imprecision. Discuss both direction and magnitude of any potential bias | Please see **Discussion, last paragraph.** |  |  |
| Interpretation | 20 | Give a cautious overall interpretation of results considering objectives, limitations, multiplicity of analyses, results from similar studies, and other relevant evidence | Please see whole **Discussion.**  S. haematobium infection investigated in this study in Mbeya region of Southwestern Tanzania revealed highly focal infection with prevalences between 0 and 16% in the different study sites. Age patterns for S. haematobium infection are similar to those reported in previous studies. Our multivariable model additionally showed, that increasing distance to water course and decreasing distance to Lake Nyasa were associated with higher odds of infection, whereas higher amount of green vegetation was assocciated with lower odds of are related to S. haematobium infection. After spatial adjustment population density became important predictor of the infection, too. **[Conclusions]** |  |  |
| Generalisability | 21 | Discuss the generalisability (external validity) of the study results | These findings and the reported pre-treatment prevalences could help to evaluate and improve ongoing and future control activities in the region, in Tanzania and elsewhere. **[Conclusions]** |  |  |
| Other information | | |  |  |  |
| Funding | 22 | Give the source of funding and the role of the funders for the present study and, if applicable, for the original study on which the present article is based | The EMINI study was funded by the European Commission (SANTE/2004/078-545/130&SANTE/2006/129-931). Helminth data collection was supported by the German Science foundation DFG (SA 1878/1-1) and the German Federal Ministry of Education and Research BMBF (01 KA 0904) with additional support from the European Commission’s Seventh Framework Programme (EC-GA no 241642). The funders had no role in study design, data collection and analysis, decision to publish, or preparation of the manuscript. |  |  |

*Give information separately for exposed and unexposed groups.

**Note:** An Explanation and Elaboration article discusses each checklist item and gives methodological background and published examples of transparent reporting. The STROBE checklist is best used in conjunction with this article (freely available on the Web sites of PLoS Medicine at http://www.plosmedicine.org/, Annals of Internal Medicine at http://www.annals.org/, and Epidemiology at http://www.epidem.com/). Information on the STROBE Initiative is available at www.strobe-statement.org.
