# Supplementary material for: Schistosoma haematobium infection and environmental factors in Southwestern Tanzania: A cross-sectional, population-based study
Source: PLoS Negl Trop Dis. 2020 Aug 24;14(8):e0008508. doi: 10.1371/journal.pntd.0008508 (PMC7446842; doi:10.1371/journal.pntd.0008508)
Supplement: S1 Table — Results of multivariable mixed effects logistic regression with site and household as random effects and with binary S. haematobium infection status as the outcome (N = 17,280). *Multivariable mixed effects logistic model with random effects for households and study sites. §Multivariable generalized additive mixed model of binomial family with spatially correlated effects base on the location of the observation, with additional random effects on households and study sites. OR = odds ratio, CI = confidence interval, SES = socio-economic status. The reference category for stratified variables is denoted by the asterisk *. (DOCX) [file pntd.0008508.s002.docx]

**Table S1:** **Multivariable association of socio-demographic factors only with *S. haematobium* infection.**

| All study sites (N=17280) | Base model non-spatial^*^ | | | Base model spatial^§^ | | |
| --- | --- | --- | --- | --- | --- | --- |
| **Covariate** | **OR** | **95% CI** | **p value** | **OR** | **95% CI** | **p value** |
| **Sex** |  |  |  |  |  |  |
| Female* | 1.00 | - | - | 1.00 | - | - |
| Male | 1.02 | 0.87 – 1.21 | 0.7769 | 1.04 | 0.89 – 1.21 | 0.6479 |
| **Age** (years) |  |  |  |  |  |  |
| below 5 | 1.17 | 0.78 – 1.77 | 0.4474 | 1.15 | 0.76 – 1.75 | 0.4985 |
| 5-15 | 7.85 | 5.88 – 10.48 | **<0.0001** | 7.66 | 5.76 – 10.17 | **<0.0001** |
| 15-25 | 5.89 | 4.33 – 8.01 | **<0.0001** | 5.78 | 4.27 – 7.82 | **<0.0001** |
| 25-35 | 1.66 | 1.13 – 2.44 | **0.0097** | 1.67 | 1.14 – 2.45 | **0.0090** |
| 35 and above * | 1.00 | - | **-** | 1.00 | - | - |
| **HIV infection** |  |  |  |  |  |  |
| No* | 1.00 | - | - | 1.00 | - | - |
| Yes | 0.65 | 0.40 – 1.05 | 0.0753 | 0.65 | 0.40 – 1.03 | 0.0681 |
| no information | 0.68 | 0.35 – 1.30 | 0.2393 | 0.71 | 0.38 – 1.32 | 0.2747 |
| **SES score** (per 1 unit) | 0.94 | 0.77 – 0.99 | **0.0325** | 0.95 | 0.84 – 1.07 | 0.3991 |

*Multivariable mixed effects logistic model with random effects on households and study sites. ^§^Multivariable generalized additive mixed model of binomial family with spatially correlated effects base on the location of the observation, with additional random effects on households and study sites. OR=odds ratio, CI=confidence interval, SES = socio-economic status. The reference category for stratified variables is denoted by the asterisk *.
