# Supplementary material for: Schistosoma haematobium infection and environmental factors in Southwestern Tanzania: A cross-sectional, population-based study
Source: PLoS Negl Trop Dis. 2020 Aug 24;14(8):e0008508. doi: 10.1371/journal.pntd.0008508 (PMC7446842; doi:10.1371/journal.pntd.0008508)
Supplement: S2 Table — *Multivariable mixed effects logistic model with random effects for households and study sites. §Multivariable generalized additive mixed model of binomial family with spatially correlated effects base on the location of the observation, with additional random effects for households and study sites. (DOCX) [file pntd.0008508.s003.docx]

**Tabel S2: Full model: Association of socio-demographic and environmental factors with S. haematobium infection.**

| All study sites (N=17280) |  |  | Full model non-spatial* | | | Full model spatial ^§^ | | |
| --- | --- | --- | --- | --- | --- | --- | --- | --- |
| **Covariate** | **N** | **% pos.** | **OR** | **95% CI** | **p value** | **OR** | **95% CI** | **p value** |
| **Sex** |  |  |  |  |  |  |  |  |
| Female* | 9194 | 4.95 | 1.00 | - | - | 1.00 | - | - |
| Male | 8086 | 5.68 | 1.02 | 0.87 - 1.21 | 0.7689 | 1.02 | 0.87 – 1.19 | 0.7959 |
| **Age** (years) |  |  |  |  |  |  |  |  |
| below 5 | 2233 | 2.15 | 1.14 | 0.76 - 1.72 | 0.5253 | 1.11 | 0.73 – 1.66 | 0.6311 |
| 5-15 | 5534 | 8.98 | 7.73 | 5.79 - 10.33 | **<0.0001** | 7.19 | 5.43 – 9.50 | **<0.0001** |
| 15-25 | 3063 | 7.61 | 5.80 | 4.26 - 7.89 | **<0.0001** | 5.48 | 4.06 – 7.38 | **<0.0001** |
| 25-35 | 2336 | 2.65 | 1.63 | 1.11 - 2.39 | **0.0131** | 1.58 | 1.08 – 2.32 | **0.0180** |
| 35 and above* | 4114 | 1.80 | 1.00 | - | **-** | 1.00 | - | - |
| **HIV infection** |  |  |  |  |  |  |  |  |
| No* | 15700 | 5.57 | 1.00 | - | - | 1.00 | - | - |
| Yes | 1209 | 2.07 | 0.64 | 0.40 - 1.03 | 0.0667 | 0.62 | 0.38 – 0.99 | **0.0432** |
| No information | 371 | 4.04 | 0.70 | 0.36 - 1.33 | 0.2731 | 0.72 | 0.39 – 1.35 | 0.3070 |
| **SES score** (per 1 unit) |  |  | 0.89 | 0.78 - 1.02 | 0.0983 | 0.90 | 0.79 – 1.01 | 0.0750 |
| **Population density** (per 1000 persons/km²) |  |  | 1.08 | 0.97 - 1.20 | 0.1710 | 1.26 | 1.09 – 1.45 | **0.0019** |
| **Elevation** (per 100 m) |  |  | 0.73 | 0.63 - 0.85 | **<0.0001** | 0.70 | 0.45 – 1.10 | 0.1208 |
| **EVI minimum** (per 0.1 units) |  |  | 0.21 | 0.11 - 0.39 | **<0.0001** | 0.16 | 0.07 – 0.35 | **<0.0001** |
| **Distance to water course** (in km) |  |  | 1.36 | 1.20 - 1.56 | **<0.0001** | 1.17 | 1.03 – 1.34 | **0.0202** |
| **Distance to Lake Nyasa** (in km) |  |  |  |  |  |  |  |  |
| below 1 | 195 | 9.23 | 5.73 | 2.09 - 15.68 | **0.0007** | 4.35 | 0.83 – 22.82 | 0.0825 |
| 1-2 | 372 | 9.14 | 4.47 | 1.90 - 10.50 | **0.0006** | 3.77 | 1.00 – 14.25 | 0.0509 |
| 2-4 | 428 | 11.92 | 4.13 | 1.91 - 8.91 | **0.0003** | 4.00 | 1.47 – 10.93 | **0.0068** |
| 4 and above* | 16285 | 4.98 | 1.00 | - | **-** | 1.00 | - | - |
| **Number of persons in household** (per person) |  |  | 1.01 | 0.98 - 1.05 | 0.5194 | 1.01 | 0.98 – 1.04 | 0.5163 |
| **Household with latrine** |  |  |  |  |  |  |  |  |
| No* | 419 | 8.35 | 1.00 | - | **-** |  |  |  |
| Yes | 16861 | 5.21 | 0.72 | 0.40 - 1.29 | 0.2696 | 0.61 | 0.36 – 1.03 | 0.0649 |
| **Rainfall** (per 100 mm) |  |  | 1.08 | 0.93 - 1.26 | 0.3257 | 0.99 | 0.65 – 1.51 | 0.9529 |
| **Slope** (per 1°) |  |  | 1.05 | 0.95 - 1.16 | 0.3106 | 0.98 | 0.87 – 1.10 | 0.7111 |

*Multivariable mixed effects logistic model with random effects on households and study sites. ^§^Multivariable generalized additive mixed model of binomial family with spatially correlated effects base on the location of the observation, with additional random effects on households and study sites.
