# Supplementary material for: Schistosoma haematobium infection and environmental factors in Southwestern Tanzania: A cross-sectional, population-based study
Source: PLoS Negl Trop Dis. 2020 Aug 24;14(8):e0008508. doi: 10.1371/journal.pntd.0008508 (PMC7446842; doi:10.1371/journal.pntd.0008508)
Supplement: S3 Table — (DOCX) [file pntd.0008508.s004.docx]

**Table S3:** ***S. haematobium* infection prevalences in children below 5 years of age.**

|  | ***S. haematobium* prevalence (children < 5 years)** | | |
| --- | --- | --- | --- |
| **Study site** | **No. of participants** | **No. of infections** | **Infection prevalence % (95% CI)** |
| Igurusi | 253 | 23 | 9.1 (6.1–13.3) |
| Itaka | 288 | 11 | 3.8 (2.1–6.8) |
| Kyela | 318 | 5 | 1.6 (0.7–3.7) |
| Mlowo | 259 | 1 | 0.4 (0.1–2.7) |
| Utengule | 226 | 7 | 3.1 (1.2–6.4) |
| Iyunga | 171 | 1 | 0.6 (0.1–4.1) |
| Ruanda | 169 | 0 | 0 |
| Isongole | 266 | 0 | 0 |
| Santylia | 283 | 0 | 0 |
| All Sites | 2233 | 48 | 2.1 (1.6-2.8) |

CI: confidence interval.
