# Supplementary material for: Schistosoma haematobium infection and environmental factors in Southwestern Tanzania: A cross-sectional, population-based study
Source: PLoS Negl Trop Dis. 2020 Aug 24;14(8):e0008508. doi: 10.1371/journal.pntd.0008508 (PMC7446842; doi:10.1371/journal.pntd.0008508)
Supplement: S4 Table — CI: confidence interval. (DOCX) [file pntd.0008508.s005.docx]

**Table S4:** **Prevalence of *S. haematobium* infection over all survey rounds of the EMINI study.**

| Survey round | Year | No. of participants | No. of infections | Infection intensity (light %, heavy %) | Infection prevalence % (95% CI) |
| --- | --- | --- | --- | --- | --- |
| 1. | 2006 - 2007 | 17280 | 914 | 89.2%, 10.8% | 5.3 (5.0 – 5.6) |
| 2. | 2007 - 2008 | 17157 | 590 | 92.5%, 7.5% | 3.4 (3.2 – 3.7) |
| 3. | 2008 – 2009 | 14994 | 355 | 97.7%, 2.3% | 2.4 (2.1 – 2.6) |
| 4./5. | 2009 - 2010 /  2010 - 2011 | 13083 | 326 | 97.5%, 2.5% | 2.5 (2.2 – 2.8) |

CI: confidence interval.

Of note, during the last two survey round years, due to logistical reasons, only half of the households from the previous surveys could be visited each year. This is the reason why the last prevalence is reported as “survey round 4./5.”.
