# Supplementary material for: Schistosoma haematobium infection and environmental factors in Southwestern Tanzania: A cross-sectional, population-based study
Source: PLoS Negl Trop Dis. 2020 Aug 24;14(8):e0008508. doi: 10.1371/journal.pntd.0008508 (PMC7446842; doi:10.1371/journal.pntd.0008508)
Supplement: S5 Table — Results of multivariable mixed effects negative binomial regression with site and household as random effects and with S. haematobium egg counts as the outcome, adjusted for socio-demographic factors. Only includes S. haematobium infected participants (N = 914). ECR = egg count ratio, CI = confidence interval, SES = socio-economic status. The reference category for stratified variables is denoted by the asterisk *. (DOCX) [file pntd.0008508.s006.docx]

**Table S5:** **Association of HIV status with *S. haematobium* infection intensity.**

| All study sites | Univariable model | | | Multivariable model | | |
| --- | --- | --- | --- | --- | --- | --- |
| **Covariate** | **ECR** | **95% CI** | **p value** | **ECR** | **95% CI** | **p value** |
| **HIV infection** |  |  |  |  |  |  |
| No* | 1.00 | - | - | 1.00 | - | - |
| Yes | 0.90 | 0.69 – 1.17 | 0.4300 | 1.68 | 0.98 – 2.90 | 0.0608 |
| no information | 0.98 | 0.68 – 1.43 | 0.9316 | 1.07 | 0.74 – 1.56 | 0.7101 |
| **Sex** |  |  |  |  |  |  |
| Female* |  | - | - | 1.00 | - | - |
| Male |  |  |  | 1.24 | 0.72 – 2.13 | 0.4361 |
| **Age** (years) |  |  |  |  |  |  |
| below 5 |  |  |  | 2.59 | 0.31 – 21.36 | 0.3773 |
| 5-15 |  |  |  | 3.83 | 1.16 – 12.64 | **0.0277** |
| 15-25 |  |  |  | 2.87 | 1.13 – 7.29 | **0.0271** |
| 25-35 |  |  |  | 1.17 | 0.51 – 2.69 | 0.7127 |
| 35 and above * |  |  |  | 1.00 | - | - |
| **SES score** (per 1 unit) |  |  |  | 0.92 | 0.81 – 1.03 | 0.1427 |

ECR= Egg count ratio, CI= confidence interval.
