# Supplementary material for: Schistosoma haematobium infection and environmental factors in Southwestern Tanzania: A cross-sectional, population-based study
Source: PLoS Negl Trop Dis. 2020 Aug 24;14(8):e0008508. doi: 10.1371/journal.pntd.0008508 (PMC7446842; doi:10.1371/journal.pntd.0008508)
Supplement: S1 Text — (DOCX) [file pntd.0008508.s007.docx]

**Details variable selection *S.haematobium* infection modelling**

A “base model” including the variables sex, age, HIV status and SES was built in STATA using mixed effects logistic regression with household and study site as random effects. These variables were included due to their relevance to schistosomiasis infection. For this model AIC was 5511.374.

The following additional candidate variables were used in the modelling:

- age (0-5 years, 5-15 years, 15-25 years, 25-35 years vs. 35 years and over)
- sex (male vs. female)
- HIV status (positive, no information vs. negative)
- socio-economic status^[[1]](#footnote-1)^ (SES, standardized, per unit)
- number of persons in household (per person)
- availability of a latrine in household (yes vs. no)
- population density (per 1000 persons/km^2)
- minimum, mean and maximum annual enhanced vegetation index (EVI, per 0.1 units)
- elevation (per 100 m)
- mean annual land-surface temperature (LST) during the night (per 1°C)
- mean annual LST during the day (per 1°C)
- mean annual rainfall (per 100 mm)
- slope of the terrain (per °)
- distance to nearest water course (in km)
- distance to lake Nyasa (0-1 km, 1-2 km, 2-4 km vs. 4 km and more)

A user-written STATA macro was used to idenfity the most parsimonious model according to the AIC. This was done in a step-by-step manner and illustrated in Figures 1 and 2. In the first step the macro estimates models including variables of the base model and each of the remaining predictors one by one (see Fig. 1). AIC for each model is calculated, too. The model with the lowest AIC is chosen (i.e. base model with elevation in Fig. 1), if the AIC remains lower then the AIC of the base model and no collinearity issues arise (thus the variance inflation factor VIF remains below 10).


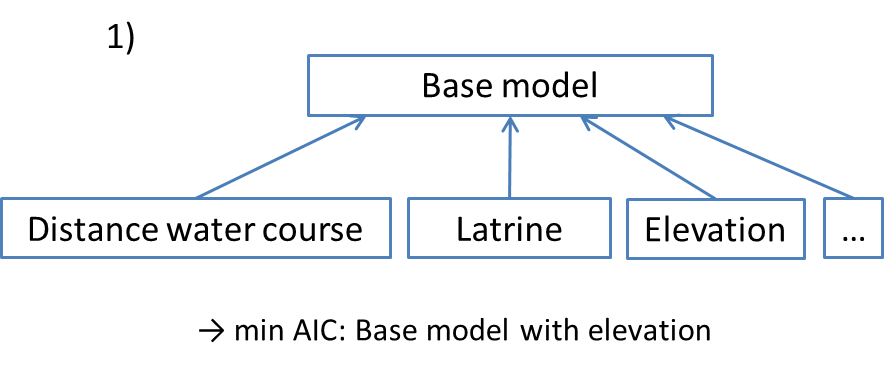


Figure 1: Illustration of the variable selection procedure according to minimal AIC: Step 1.

In the next step (Fig. 2) we started with the modified model identified in step 1 (i.e. base model with elevation) and estimated new models by including all explanatory variables one by one. The model with minimal AIC was chosen (elevation, Fig. 1), if its AIC remained lower than the AIC of the model from the previous step and the VIF remained below 10. This way variables were included/excluded one by one until the model with the most parsimonious AIC was found.


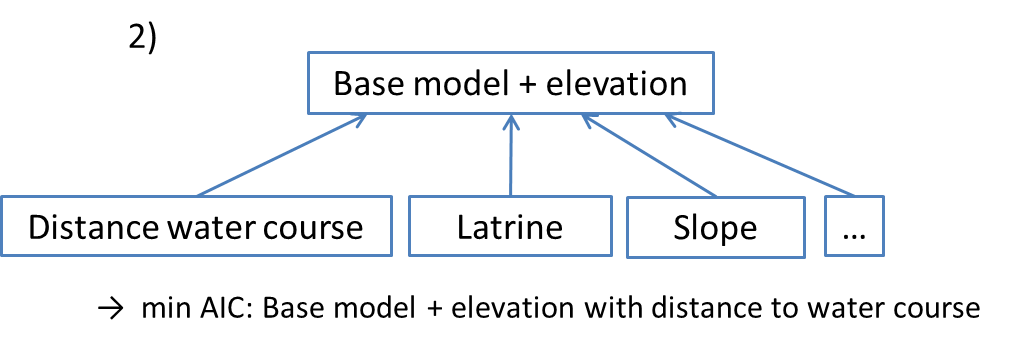


Figure 2: Illustration of the variable selection procedure according to minimal AIC: Step 2.

Table 1 shows the AICs and the order of the variable inclusion.

| **Order of inclusion** | **Variable** | **AIC** |
| --- | --- | --- |
|  | Base model | 5511.374 |
| 1 | Elevation | 5488.093 |
| 2 | Distance to water course | 5468.282 |
| 3 | EVI minimum | 5458.153 |
| 4 | Distance to lake | 5445.770 |
| 5 | Population density | 5445.718 |

LST day and night were excluded due to collinearity with elevation. Number of persons in household, availability of a latrine, rainfall and slope were excluded due to increasing AIC.

1. For the calculation of the SES the presence of latrine in the household was excluded, since latrine availability was included as its own variable. [↑](#footnote-ref-1)
